# Wearable Sensors for Monitoring of Cigarette Smoking in Free-Living: A Systematic Review

**DOI:** 10.3390/s19214678

**Published:** 2019-10-28

**Authors:** Masudul H. Imtiaz, Raul I. Ramos-Garcia, Shashank Wattal, Stephen Tiffany, Edward Sazonov

**Affiliations:** 1 Department of Electrical and Computer Engineering, The University of Alabama, Tuscaloosa, AL 35487, USA; masudul4145@gmail.com (M.H.I.); raul.iramosg@gmail.com (R.I.R.-G.); swattal@crimson.ua.edu (S.W.); 2 Department of Psychology, University at Buffalo, The State University of New York, Buffalo, NY 12246, USA; stiffany@buffalo.edu

**Keywords:** cigarette smoking, ECG, IMU, respiration, RIP, signal processing, smoke exposure, wearable sensor

## Abstract

Globally, cigarette smoking is widespread among all ages, and smokers struggle to quit. The design of effective cessation interventions requires an accurate and objective assessment of smoking frequency and smoke exposure metrics. Recently, wearable devices have emerged as a means of assessing cigarette use. However, wearable technologies have inherent limitations, and their sensor responses are often influenced by wearers’ behavior, motion and environmental factors. This paper presents a systematic review of current and forthcoming wearable technologies, with a focus on sensing elements, body placement, detection accuracy, underlying algorithms and applications. Full-texts of 86 scientific articles were reviewed in accordance with the Preferred Reporting Items for Systematic Review and Meta-Analyses (PRISMA) guidelines to address three research questions oriented to cigarette smoking, in order to: (1) Investigate the behavioral and physiological manifestations of cigarette smoking targeted by wearable sensors for smoking detection; (2) explore sensor modalities employed for detecting these manifestations; (3) evaluate underlying signal processing and pattern recognition methodologies and key performance metrics. The review identified five specific smoking manifestations targeted by sensors. The results suggested that no system reached 100% accuracy in the detection or evaluation of smoking-related features. Also, the testing of these sensors was mostly limited to laboratory settings. For a realistic evaluation of accuracy metrics, wearable devices require thorough testing under free-living conditions.

## 1. Introduction

Worldwide, tobacco use is a major risk factor for disease and death. Tobacco dependence, which is classified in the International Classification of Diseases (ICD-10) [1], causes several types of pulmonary and cardiovascular illness (emphysema, chronic bronchitis, heart attacks and strokes), lethal cancers (lung, colorectal, mouth, larynx, liver, cervix, etc.), and is known to affect the reproductive and immune systems [2,3,4]. It also increases the chance of severe health issues like diabetes, duodenal ulcers, loss of appetite, atherosclerosis, age-related macular degeneration and vision loss, premature birth and even miscarriages in pregnant women [5]. Cigarette smoking is the predominant form of tobacco use.

In 2017, an estimated 47.4 million U.S. adults (19.3%) were reported as using tobacco products, including cigarettes (14.0%; 34.3 million); cigars, cigarillos, or filtered little cigars (3.8%; 9.3 million); electronic cigarettes (e-cigarettes) (2.8%; 6.9 million); smokeless tobacco (2.1%; 5.1 million); and pipes, water pipes, or hookahs (1.0%; 2.6 million) [6]. Research shows that the lifespan of cigarette smokers is generally reduced by 13–14 years [7]. Also, the toxicants in second-hand smoke (smoke inhaled by people in the surroundings of tobacco smokers), such as carbon monoxide (CO), tobacco-specific nitrosamines (TSNA), formaldehyde (CH_2_O), and hydrogen cyanide (HCN), have a deadly impact upon chronic obstructive pulmonary diseases (COPD) and asthma [8]. According to the United States Centers for Disease Control and Prevention (CDC, Atlanta, GA, USA) estimation, every year about 480,000 deaths are related to first-hand smoking (direct smoke inhalations) and 41,000 to second-hand smoke [9]. The World Health Organization (WHO) has estimated that annual deaths related to smoking will be 10% (more than 8 million people per year) by 2030 worldwide [10]. Of those deaths, 75% will be in low- and middle-income countries. There are also substantial economic consequences of smoking. In the United States alone, an annual cost of more than $300 billion, including $170 billion for direct medical care and $156 billion in lost productivity, is generated by 34.3 million adult smokers [11].

Despite these statistics, “smoking cessation is often hindered by the low perceivability of health risks and the unawareness of habits in day-to-day life” [12]. Data from the National Health Interview Survey (NHIS [13]) suggest that 68% of smokers are interested in quitting, and 85% have attempted quitting at least once in their lifetime [14,15], averaging 4 quitting attempts [16], with 70% of these quit efforts failing eventually [17]. Although there are numerous treatments available to help people quit smoking [18,19,20,21,22,23,24], the overall success rates of smoking cessation interventions are low. A critical starting point for these smoking cessation methods is the collection of information on the smoking habits of the individual. Self-reports of the ‘number of cigarettes smoked’ were among the first accepted measures of this information [25]. These approaches include self-report history methods such as 24-h/7-day retrospective smoking recall [26], immediate logging of cigarettes after consumption [27], and instrumented methods, such as ecological momentary assessment (EMA [28]). Self-report methods have improved in convenience and duration with the increased use of smartphones [29]. Clinical interventions (nicotine patches [30], personal counseling [31,32,33,34], etc.) mostly depend upon these self-report methods to understand smoking habits and estimate the degree of smoke exposure. However, these methods cannot capture detailed smoking metrics (the depth of inhalations, duration of smoke holding, the number of puffs-smoke intake per cigarette, the duration, or other aspects of smoke exposure [35]), which can support effective interventions and lapse monitoring. Also, as self-report approaches rely heavily on the user’s recall and impose a burden on the smokers [25], the accuracy of these self-reports is generally limited by memory biases and intentional or unintentional misrepresentations or underreporting [36].

During the past decade, a wide range of technology-driven smoking assessments has been investigated, such as expired CO monitoring [37,38], biomarkers [39] and image processing [40,41,42]. However, no usable pattern of inhalations or smoking habits can be drawn from expired CO- or biomarker-based approaches [38]. A commercial handheld monitoring device, the Clinical Research Support System (CReSS) [43], was developed to acquire and store behavioral information about smoking in the natural environment. However, the use of this ‘smoke-through’ CReSS device may affect the pattern of inhalations in many smokers due to its obtrusiveness and large size [44]. Moreover, the ability of this device to capture all instances of smoking does demand that the people being monitored smoke all their cigarettes through the device—not all smokers are compliant with these instructions. Surveillance camera-based imaging methods require the installation of video cameras in all possible smoking locations, which is not feasible at the community level [45].

Recently, wearable sensors [46] have drawn attention as a potential solution to the problem of the passive detection of cigarette smoking and smoke exposure. Wearable sensors are lightweight, mobile, convenient, with the ability for ‘collecting data anytime, anywhere and often’ [47]. These devices are composed of varying sensing modalities, such as electrical, inertial (individual or multi-axis combinations of precision gyroscopes, accelerometers, magnetometers), acoustic, etc. Some approaches have used a combination of sensors. However, no single wearable method has been found to be 100% accurate for detecting smoking events in all circumstances, isolating puffs and smoke inhalations, or evaluating the metrics of smoke exposure. Some technologies suit certain environments, while others fail to provide good results in the same context. The sensor responses are often influenced by ambient factors, such as motion and clothing. To date, no in-depth survey or comparison (trade-off) study of these approaches has yet been performed highlighting the advantages and limitations of sensing technologies or their applicability in naturalistic settings. Also, there has been little evaluation of the underlying detection algorithms and their comparative accuracy.

This review is intended to provide a systematic evaluation of state-of-the-art wearable sensors for monitoring cigarette smoking in free-living conditions. The primary focus of this review is an up-to-date summary of recent novel approaches, individual and multi-sensor combinations, body locations, processing of sensor signals, detection algorithms and assessments of comfort. To cover the full range of the monitoring systems of cigarette smoking in this survey, research publications and commercially available sensor systems were thoroughly studied, and a total of 314 papers (without duplication) were found related to these topics. Following the application of inclusion and exclusion criteria, 108 papers were selected for a full-text review.

The paper is organized as follows. First, the methodology of the systematic review is presented in Section 2, along with the specification of the research questions (RQ). Section 3 and Section 4 present the detailed exploration of these research questions, with the identification of the behavioral and physiological manifestations of cigarette smoking (Section 3), and the evaluation of wearable sensing technologies (Section 4). Section 5 discusses the challenges and potential research focus in the field of automated monitoring. Section 6 provides a summary of the review.

## 2. Review Methodology

This review of the monitoring of cigarette smoking in free-living conditions was conducted according to the Preferred Reporting Items for Systematic Review and Meta-Analyses (PRISMA) [48] guidelines. The systematic search procedure was primarily set by three authors (MHI, SW and ES), and was executed with the assistance of the remaining authors (RRG and ST). Two authors (MHI and SW) independently screened the titles and abstracts of the publications retrieved through the database search; then one author, (MHI), carried out a full-text review of all relevant studies. This methodology used the following processes:

### 2.1. Identifying Research Question

Three research questions (RQs) were chosen to guide this systematic review:

(1) RQ1. *What were the behavioral and physiological manifestations of cigarette smoking targeted by wearable sensors for the detection of cigarette smoking?* The answer to this question will help in understanding the rationale behind the implementation of these sensors and aid in investigating the impact of their placement on the body.

(2) RQ2. *What were the sensor modalities employed to detect these manifestations?* The answer to this question will identify key sensor modalities used in wearable sensors for monitoring cigarette smoking.

(3) RQ3. *What were signal processing and pattern recognition methods applied to the sensor signals? Further, how was the performance of the sensors evaluated, and what accuracies were achieved?* The answer to this question will help in analyzing the adequacy of current approaches and help in identifying the research gaps in current methodologies.

### 2.2. Source of Studies

Exhaustive electronic searches for relevant literature were performed across seven repositories: PubMed, Google Scholar, Science Direct, Wiley Online Library, ACM Digital library, MDPI and IEEE Explore from inception through to 30 September 2019.

### 2.3. Search Strategy

For the purpose of this review, the devices that are capable to be worn on or attached to the body, and are capable of providing usable data to the wearer, were broadly defined as wearable technology. To cover all wearable technologies intended for both smoking research and general use, the following ‘free-text search terms’ and their alternative spellings and plurals were used: ‘wearable sensors’, ‘smoking detection’, ‘monitoring of smoking’, ‘inhalation assessment’, and ‘smoking cessation’. The search results were also strictly restricted to the English language. References from the selected primary full-text articles were further analyzed for relevant publications. All articles were subsequently grouped based upon common themes, such as assessed wearable devices, methods, key results, study setting and publication type. The selection was further narrowed by applying the eligibility criteria described in Table 1. Articles fulfilling the inclusion criteria were considered in this review, and those fulfilling the exclusion criteria were filtered out.

### 2.4. Results

**Figure 1:**
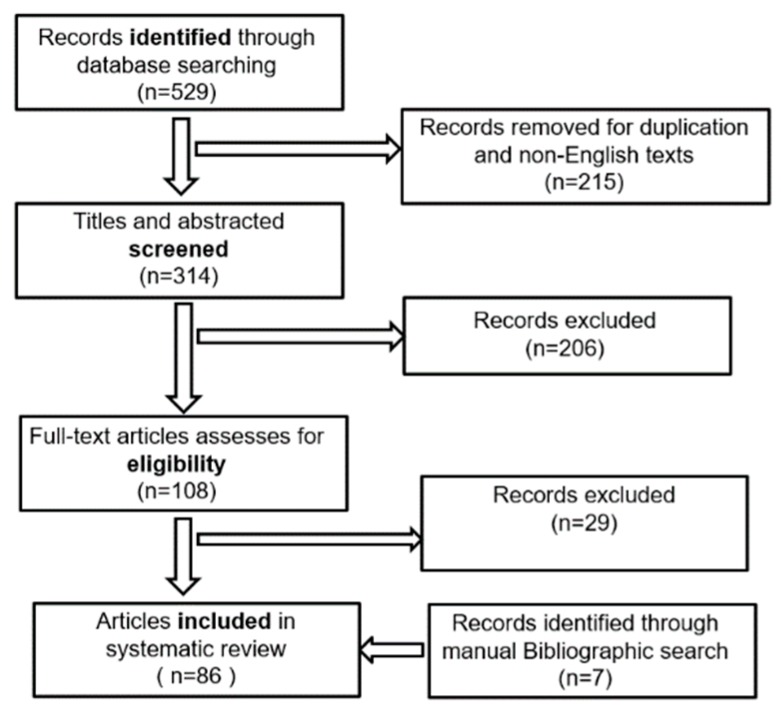
Flow diagram depicting the systematic review strategy.

A total of 314 publications, identified through the database search, were set for the title and abstract screening; of these, 108 articles were selected for the full-text review. However, 29 failed to satisfy the eligibility criteria, and were excluded. A manual bibliographic search also identified seven additional publications qualified to be included for the full-text review. Thus, 86 publications ultimately fulfilled the eligibility criteria for this review. Figure 1 illustrates the methodology and results of the review process.

A total of 31 publications were found (summarized in Table 2) describing the classical methods for smoking detection, including smoking self-reporting, pathological and technological methods such as CO- and biomarker-based measurement, surveillance video camera-based approaches, etc. As the key focus of this review was the detection of smoking employing wearable sensors, the detailed evaluation of these classical methods was omitted here. For the same reason, another four papers that employed smartphones to monitor the smoking habits of the user were omitted.

## 3. Behavioral and Physiological Manifestations of Cigarette Smoking

The philosophy behind the implementation of wearable sensors in smoking detection requires a thorough comprehension of the cigarette smoking process. The frequency or pattern of cigarette smoking generally varies between individuals or brands of the smoked cigarette; however, a few similarities between behavioral and physiological phenomena are always present [49,50]. An average smoker smokes a cigarette in 4–8 min [51] with 8–16 puffs [52]. The process starts with the removal of a cigarette from a packet, generally using fingers (sometimes using the combination of teeth and lips), putting the filtered end in the mouth, and lighting up. The number of consumed cigarettes may be tracked from the cigarette packet or holder, if it is instrumented accordingly. Also, smokers usually carry a personal lighter or match, and use it to light their cigarettes. The frequency of cigarette consumption can be identified from cigarette lighting events [12].

Once the cigarette is lit, smokers inhale and move their hands away from the mouth. This step is repeated throughout the smoking session. During puffs, the smoking hand stays vertically close to the mouth. Specifically, for inhalations, the fingers holding the cigarette reach closer to the lips and the wrist moves close to the chest. The positioning of these body parts can be used as a potential indicator of smoking events [53].

When people pull their hands closer to their mouths (from the rest) for puffing, they need to work against the pull of the acceleration due to gravity. When the hand remains stationary, close to the lips, this gravitational acceleration stays constant. When the hand returns after puffing, it works along with gravity. Smoking puffs can be identified from these hand-to-mouth gestures (HMGs). Rotations or angular motions of the smoking hand during a puff sequence also have distinguishing features. These rotations occur in a certain direction when the hand moves towards the mouth, and in the opposite direction when the hand moves away from the mouth. These rotations can also indicate smoking events [54].

**Figure 2:**
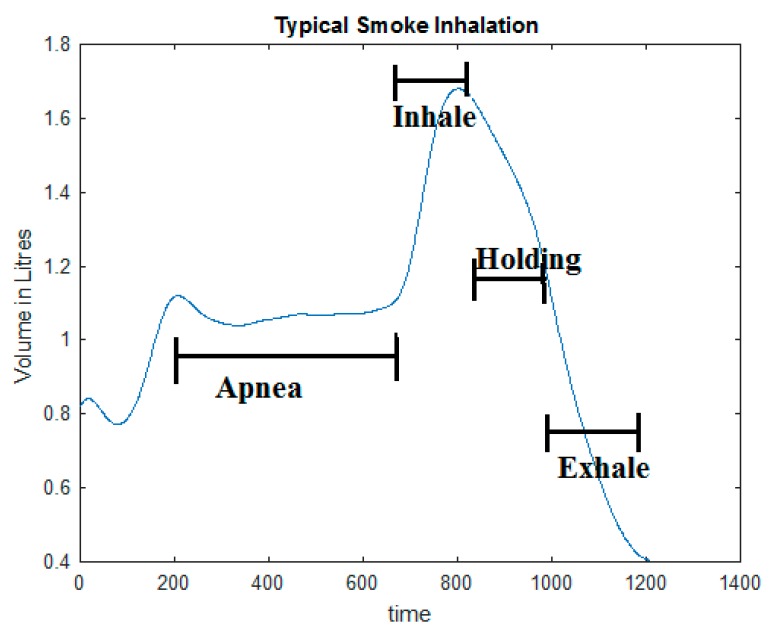
An illustration of a smoking-specific respiration pattern (horizontal axis: Time in milliseconds, vertical axis: Breath volume).

Regarding smoke inhalations, smokers generally do not inhale during cigarette lighting [55], and inhale a very small amount during the initial puffs. To avoid irritation in the throat in the initial puffs, some smokers briefly hold the smoke in their mouth. Major smoke inhalations are done either by deep breathing, and occasionally by ‘Frenching’ (pushing some smoke back into the air without exhaling completely, and inhaling it through the nose—also referred to as a ‘Chinese Drawback’) [55]. A smoke inhalation can be summarized as a sequential process of: (a) A cessation of normal air-intake (breathing apnea) during cigarette holding; (b) A sharp increase of tidal volume and airflow due to smoke inhalation into the lungs; (c) Occasionally a brief period of smoke holding in the lungs, and; (d) A slow or forced exhalation, either through nose or mouth [56]. This characteristic respiration pattern may also be an indicator of smoking. Figure 2 illustrates a typical smoke inhalation in terms of changes in the lung breath volume.

There is a significant difference between the acoustic properties of a smoking breath and a non-smoking breath. By characterizing these differences in a non-invasive way, it may be possible to detect the smoking episodes [57].

Some instantaneous changes in the physiological parameters of the smoker (such as blood pressure [58], and heart rate [59], etc.) also occur during smoking. These parameters, if characterized correctly, may help identify smoke inhalations.

Again, hand-oriented smoking activities (such as cigarette lighting, hand to mouth gestures and a cigarette holding between puffs) may require smokers to frequently look at their hands. An egocentric camera, such as a camera positioned on the head or chest of the person, naturally approximates the visual field of the camera wearer, and offers a valuable perspective to understand the smoking activity and their context in a naturalistic setting.

A total of 51 research studies employing wearable sensors of different modalities addressing these behavioral and physiological manifestations associated with smoking have been reported in the last decade. These approaches have been validated on a number of smoker subjects. Table 3 provides a brief summary of these publications.

## 4. Evaluation of Sensing Methodologies

### 4.1. Individual Sensor Approach

A brief evaluation of wearable sensor modalities is provided below, with the sequential responses to RQ2 and RQ3, grouping by their accomplishments in detecting smoking-related features.

#### 4.1.1. Detection of Smoking Frequency from Cigarette Lighting

Sensor modalities: Unlike matches, the cigarette lighter allows for further instrumentation. Hence, a data logging system and a real-time clock may be integrated into the personal lighter to allow measurements of the frequency of cigarette consumption from ignition counts and recordings of accurate timestamps of the smoking episodes.

**Figure 3:**
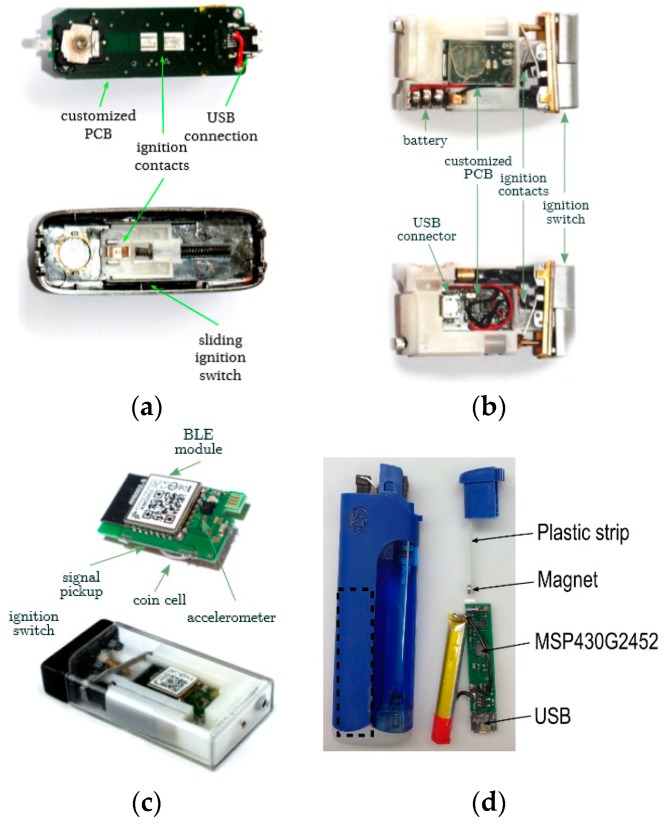
The instrumented lighters: (**a**) UbiLighter v1; (**b**) UbiLighter v2; (**c**) UbiLighter v3; (**d**) the Personal Automatic Cigarette Tracker (PACT) lighter (image source: [60,61]).

Embedding sensors in commercially available cigarette lighters was introduced by Scholl et al. [12] to detect lighting events prior to cigarette smoking. Three successive versions of an augmented lighter named UbiLighter [60] were introduced with an interfaced microcontroller configured to record lighter ignition events (with timestamps) in its internal memory. UbiLighter v1 (Figure 3a) was a modified electronic lighter in which closing electric contacts (by a slide down switch) triggered an interrupt to the microcontroller and heated the electric coil to ignite the lighter. UbiLighter v2 (Figure 3b) was a modified gas lighter where the microcontroller was connected to contacts that closed upon a press on the ignition button. UbiLighter v3 (Figure 3c) was based on a piezo-ignition concept where a copper contact, connected to a microcontroller, was placed in the vicinity of piezo-element to pick up a voltage when the lighter was ignited. Imtiaz et al. [61] proposed another augmented gas-lighter approach (shown in Figure 3d), employing a low-power Hall Effect sensor. The button of this lighter was instrumented by a magnet that triggered proximity events in the Hall Sensor upon button press. The details of these lighters are tabulated in Table 4. Among other commercially available electronic lighters, Quitbit [62], an internet-enabled special e-lighter, is similarly capable of measuring cigarette counts using integrated electronics in its heating coil.

Signal Processing and Pattern recognition: The recorded timestamps from lighter ignition events have been used as indicators of cigarette consumption with minimal processing. In some of these approaches, closely spaced lighting events (a few seconds apart) were merged and considered as a single lighting event for two purposes: First, to debounce the ignition contact, and second, to filter incidents of the multiple ignitions sometimes needed to light a cigarette.

Evaluation: A bench test on the accuracy of lighter records was reported by Imtiaz et al. [61] with a root-mean-squared error (RMSE) value of 0.68 sec for the timestamp of ignition events over 168 h (one week) vs. the true timestamp. Validation (field) study details of these instrumented lighters are also tabulated in Table 4.

#### 4.1.2. Detection of HMG Preceding Smoking Based on Hand to Mouth Proximity

Sensor modality: Specific hand-to-mouth gestures of the arm during smoking have successfully been detected using Radio Frequency (RF) proximity sensors [53,61].

**Figure 4:**
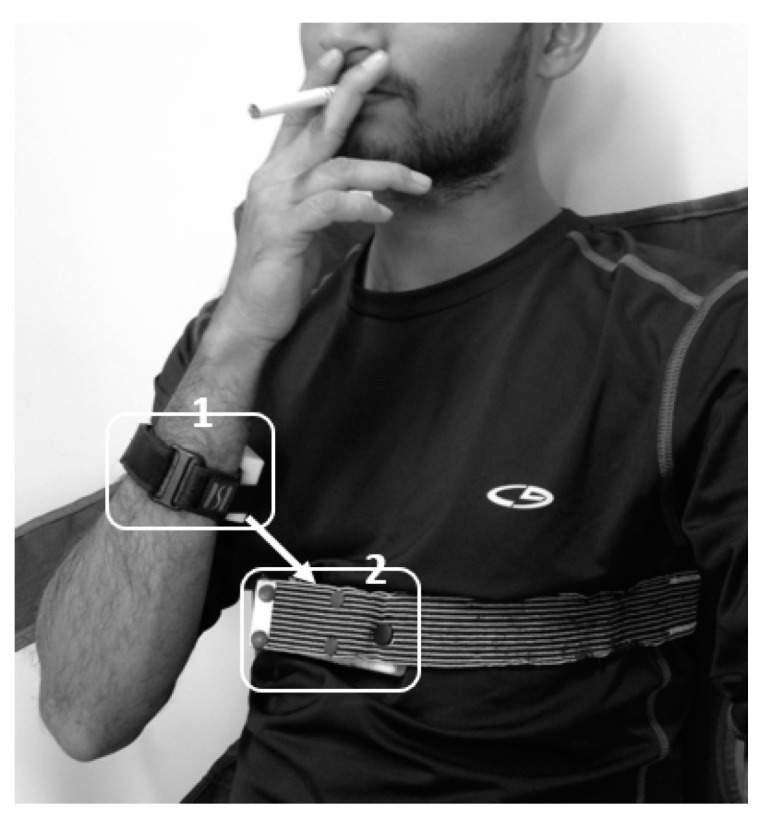
The concept of proximity sensor: Closeness of hand to mouth, i.e., the closeness of module 1 to module 2.

These electrical RF proximity sensors detected hand-to-mouth gestures preceding smoke puffs utilizing an ∞–shaped directional propagation pattern of rectangular loop antennas. This sensor used two battery-powered circuits. In the implementation [45], a small, low power RF transmitter (125 kHz) was placed onto the wrist or the inner side of the lower arm of the subject’s dominant hand, and a large receiving antenna was attached to the chest. A rectified proximity signal proportional to the distance between the transmitter–receiver antennas was generated employing a conditioning electronic circuit. In Scholl and van Laerhoven [54], another proximity sensor was proposed employing a miniature antenna at both transmitter and receiver ends (Figure 4). In this approach, the receiving antenna and embedded electronics were placed at the center of the thoracic area instead of a vest pocket (employed by Wu et al. [45]). A comparison of these two approaches is provided in Table 5.

Signal Processing and Pattern recognition: The sensor signal recorded from the RF proximity receiver was pre-processed by Imtiaz et al. [61] by applying an average Gaussian filter of 50 points; however, no smoothing methods were explicitly mentioned by Wu et al. [45]. The signals were marked as a valid hand-to-mouth movement if the amplitude was greater than the threshold of 100 mV [45] and 70 mV [61], well above their mean noise amplitudes of 90 mV and 7.31 mV respectively. A pattern recognition approach was reported by Lopez-Meyer (2013) [63], as an extension of the work done by Wu et al. [45], where the thresholds on amplitude, duration and time separation of hand gestures were empirically set for artifact rejection and gesture merging.

Evaluation: The amplitude and duration of HMGs preceding smoking were measured by Wu et al. [45]. The average gesture was found to be 3.78 (± 5.42) sec for smoking, and 6.82 (± 21.08) sec for non-smoking activities. Similarly, the average amplitude was found to be 81 (± 21.5) % and 49.3 (42.0) % of maximum amplitude, respectively. In a study on twenty subjects performing a variety of different activities in the lab [63], the RF proximity sensor demonstrated a high recall of 0.90 in detecting hand-to-mouth gestures that precede smoking puffs. In a statistical test of artifact rejection (t-test with 95% confidence interval), smoking while sitting and smoking while standing achieved a p-value of 0.000 and 0.004, respectively. Recall before and after artifact rejection was also reported to be 0.09 and 0.30, respectively.

#### 4.1.3. Detection of Smoking Events and Associated HMGs Based on Linear and Angular Movements of the Hand

**Figure 5:**
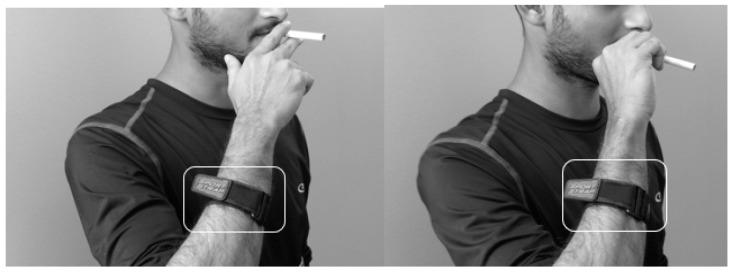
An instance of inertial sensor implementation on the dominant hand of smoking for detecting smoking hand gestures.

Sensor modality: Inertial Measurement Units (IMUs) that measure the inclination of the smoking hand [64], i.e., the transitions of arm/wrist positions or the angular/linear velocity of the hand during smoking, have been used for the detection of the hand to mouth gestures (Figure 5). Table 6 provides a summary of inertial sensor systems (individual or combination of accelerometer, gyroscope or magnetometer) employed in smoking research.

Recently, IMUs embedded in smartwatches have been employed as substitutes for wrist worn IMUs. Cole et al. (2017) [65] and Cole et al. (Dec. 2017) [66] proposed methods to employ the accelerometers of the Apple Watch (v.2.1) with the PowerSense iOS application installed on a nearby smartphone. This same study described the alternative use of Pebble’s Time Steel smartwatch with the AccelTool application. In the work by Shoaib et al. [67], the 6D IMU of a smart watch (LG Watch R). and by Añazco et al. in [68] a 6D IMU of MbientLab, were paired with smartphones and developed smoking detection applications. SmokeBeat is a similar commercial platform [69]. Further, StopWatch [70] used a standalone Android watch (LG G-Watch) to eliminate the need for a smartphone for data processing.

Signal Processing and Pattern Recognition: The first step towards processing inertial sensor data was the filtering of high-frequency noises embedded in the raw signal (although the process was not explicitly mentioned in all algorithms). Table 7 provides a brief overview of the signal processing approaches (including pre-processing) of the reported smoking detection algorithms.

Employing 3D custom IMUs, Scholl and van Laerhoven [54] detected smoking events and Tang et al. [64] both puff and smoking events by applying the simple Gaussian-based and random forest classifiers used by Scholl and van Laerhoven [54] and Tang et al. [64], respectively. Bhandari et al. [71] presented a KStar classifier (from Weka Toolkit with default parameters) on 3D IMU data to detect smoking events. From the 6D IMU signal seen in the work by Raiff et al. [72], the authors applied an SVM-based learning method followed by an edge-detection algorithm to detect both smoking events and inter-puff-intervals. Lu et al. [73] developed a Random Forest-based classifier for detecting smoking events with concurrent and confound activities. Parate et al. [74] preprocessed a 9D IMU signal as a quaternion format [75] and a probabilistic model, combining the random forest and conditional random field classifier to detect both smoking events and puffs.

In this algorithm, a relative trajectory computation method was applied to the quaternion data to discriminate, recognize and classify the different types of hand gestures.

Smart watch based approaches were reported by Cole et al. (2017) [65] and Akyazi et al. [76] employing 3D IMU sensors, and Shoaib et al. [67] and Skinner et al. [70] employing 6D sensors. To identify smoking events, these approaches applied various methods, such as artificial neural network-based classification as in Cole et al. (2017) [65] and Añazco et al. [68], a combination of Cross-word Reference Template algorithm and Dynamic Time Warping with Akyazi et al. [76], a two-layer hierarchical lazy based classification by Ramakrishnan et al. in [59], and two decision tree-based classification by Skinner et al. [70]. A detailed review of current smartwatch-based smoking detection methods is presented by Parate and Ganesan [77].

Evaluation: Table 8 provides an overview of the validation studies of the wrist IMUs. Most of these validation approaches were limited to controlled laboratory settings, only Scholl and van Laerhoven [54], Parate et al. [74] and Imtiaz et al. [61] tested subjects under free-living conditions. The study reported by Skinner et al. [70] employed the highest number of participants (38) for validation, whereas only two participants were involved in the study reported by Cole et al. (2017) [65]. Unlike other studies, the studies reported by Echebarria et al. [57], Raiff et al. [72] and Parate et al. [74] applied multiple IMUs on different body positions and identified hand to mouth gestures related to puffs as well as smoking sessions. The highest detection accuracy was reported by Parate et al. [74], employing 9D IMUs on the wrist and elbow of the dominant hand of smoking. Here, the F1-score was 0.85 and the precision and recall were 0.95 and 0.81, respectively. The minimum accuracy was reported by Scholl and van Laerhoven [54], with a precision of 0.51 and a recall of 0.70 in detecting smoking events.

#### 4.1.4. Detection of Smoking and Puffs Based on Respiratory Signals

**Figure 6:**
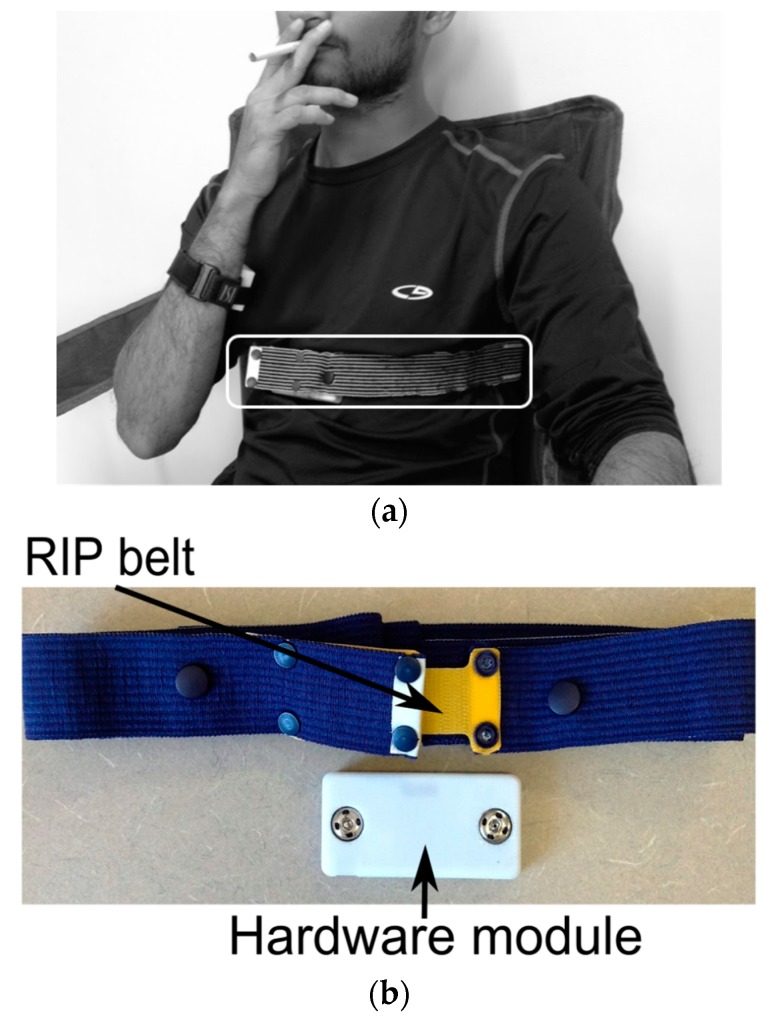
(**a**) RIP breathing sensor on chest; (**b**) sensor components.

Sensor modality: Among different lung function measurement approaches [78,79,80,81,82], at present, only Respiratory Inductance Plethysmography (RIP) [83] technology has extensively been employed to identify smoking-specific breathing patterns. An example of RIP breathing sensor implementation is provided in Figure 6. Electrical Bio-impedance (BEI) Measurement-based breathing measurement has also been introduced in Imtiaz et al. [61]. The RIP sensor, which consists of a conductive thread or wire sewn, has current applied to a conductive loop, and an opposing magnetic field is generated with strength proportional to the loop’s area, according to Lenz’s law [84]. The RIP belt, when placed around the abdominal or thoracic area, obtains respiration signals by measuring changes in the belt’s inductance caused by contraction and expansion of the lungs. This measurement is independent of the tension in the band, and not susceptible to any trapping of the RIP band to the body (unlike piezoelectric sensors [85]) or associated artifacts. Also, the bands are normally made of elastic materials, so they exert comfortable pressure and do not interfere with breathing patterns.

RIP sensors have been extensively explored to characterize the inhalation patterns associated with smoking. Initial studies [86,87,88] were performed only in laboratory settings (with thoracic and abdominal elastic respiratory bands) with bulky instrumentations and a computer-oriented Respitrace^®^ RIP module [89]. Sazonov et al. [90] conducted a laboratory study employing a commercially available, portable RIP module fed to a commercial acquisition module. The module was kept in a vest pocket of the participant and captured respiration patterns from two bands. Ali et al. [91] successfully employed the single RIP band of the AutoSense sensor suite [92] over the thoracic area to mimic the breathing pattern. Similarly, in Imtiaz et al. [61], a single RIP band was implemented by sewing the device at chest level on a T-shirt to maintain higher calibration stability [93]. A summary of wearable RIP sensor systems is presented in Table 9.

The principle of Bioimpedance measurement is significantly different from the RIP sensor. When an alternating current pass through biological tissue, the tissue impedes the flow and causes a phase shift between the sinusoidal current and the sinusoidal voltage. Using Ohm’s law, the tissue impedance can be passively calculated from the magnitude of this signal and the phase shift that is generated. Generally, high-frequency signals (20~100 kHz) can pass through the cell membrane and enable the measurement of dynamic parameters, such as intracellular and extracellular impedances. Following this principle, equivalent changes in impedance due to the modification of tidal volume can be measured by placing electrodes close to the armpits with external high-frequency excitation. This tidal volume corresponds to the amount of air flowing into and out of the lungs, which eventually mimics the instantaneous respiration waveform. Such a bio-impedance measurement was proposed by Wang et al. [94] to capture the respiration pattern from the thorax area. The study [61] introduced a bio-impedance measurement sensor into the smoking study. Here, ADS1292R, a commercially available BioZ measurement chip, was interfaced with a microcontroller to sample and record breathing at 1 kHz.

Signal Processing and Pattern Recognition: An overview of smoking detection approaches employing respiratory signals is provided in Table 10. Preprocessing of these sensor signals were necessary for the de-noising and removal of motion artifacts. The first attempt to analyze these de-noised breathing signals associated with smoking was the automatic breath segmentation [95]. In this approach, a simple breathing peak detection approach was employed for the identification of the beginning and end of a breath segment from the tidal volume (the average between the signals received from Thoracic and Abdominal breathing bands).

Recognition of daily activities that included smoking was also performed through Ramos-Garcia et al. [96] employing left-to-right hidden Markov models on tidal volume and airflow signals (the first derivate of tidal volume). A combination of a supervised and semi-supervised support vector model was proposed in Ali et al. [91] to further classify the respiration cycles obtained from a single band RIP signal into puff or non-puff events. Senyurek et al. [97] presented a Convolutional Neural Network (CNN)- and Long-Term Short Memory (LSTM)-based approach to detect smoke inhalations from RIP breathing signal and compared with a traditional machine learning (SVM)-based classifier.

In a paper under submission [98], the traditional metrics of puff duration, inhale-exhale cycle duration, smoke holding duration, inter-puff interval and novel Respiratory Smoke Exposure Metrics (RSEMs), such as inhale-exhale cycle volume and inhale-exhale volume over time were computed from the RIP breathing signal.

In the study by Imtiaz et al. [61], the generated respiration waveforms of BEI and RIP were only statistically compared with the spirometer measurements. However, BEI based method was not individually tested for the detection of smoking events.

Evaluation: Table 10 provides a summary of validation studies oriented to RIP sensors. These validations were only performed in a controlled laboratory environment with 20 subjects in Lopez-Meyer and Sazonov [95] and Ramos-Garcia, Tiffany and Sazonov [96] and 10 subjects in Ali et al. [91] mimicking daily activities. The algorithm used by Lopez-Meyer and Sazonov [95] generated an F1-score of 0.62 in identifying smoking among daily activities, whereas the algorithm used by in Ali et al. [91] found a further accuracy of 0.91 in identifying puff events. In the presence of confounding events such as stress, speaking and writing, the algorithm used by Ali et al. [91] achieved an accuracy of 0.86 in identifying smoking puffs. Also, unlike Lopez-Meyer and Sazonov in [95] and Ramos-Garcia, Tiffany and Sazonov in [96] the algorithm used by Ali et al. [91] was based on respiratory signals captured by a single band RIP sensor. In the smoking event detection by Senyurek et al. [97], the authors showed that this CNN-LSTM approach achieved an F1-score of 0.72 in leave-k-subject-out-cross-validation method whereas the classical SVM approach scored 0.63.

For puff duration, the proposed RSEM algorithm of a paper under submission [98] achieved interclass correlations (ICCs) of 0.85 and 0.87 and Pearson’s correlations of 0.97 and 0.77 with video observation and CReSS, respectively. Similarly, for the inhale-exhale duration, an ICC of 0.84 and Pearson’s correlation of 0.81 was obtained with video observation. The results suggest that the breathing signal may be used to compute smoke exposure metrics.

From the spirometer SVC test in Imtiaz et al. [61], the mean cross-correlation coefficient was obtained as 0.5438 between the bio-impedance and spirometer signal and 0.6275 between the RIP and spirometer. These BEI and RIP signals were also shown to be similar while the smokers performed multiple activities: standing, walking, resting idly in a chair, eating, and smoking cigarettes.

#### 4.1.5. Detection of Smoking Events Based on Acoustic Signals

**Figure 7:**
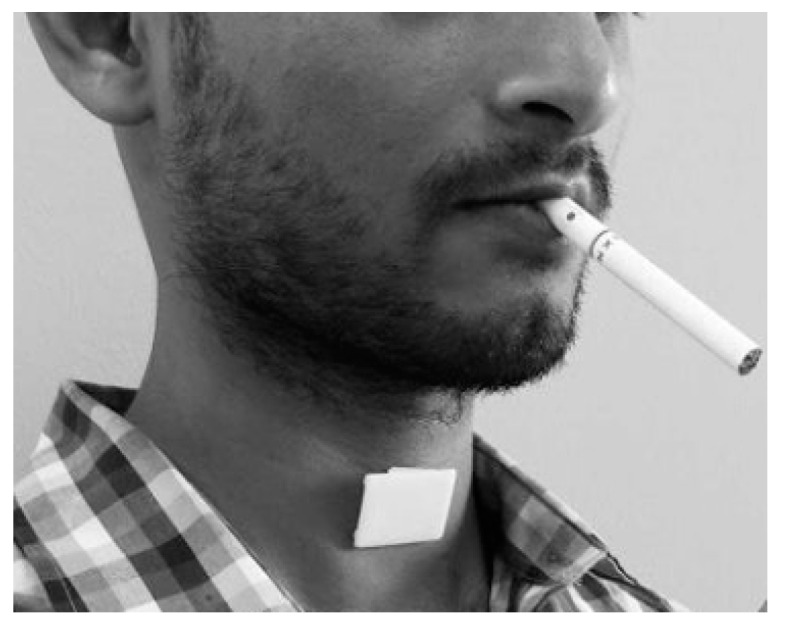
Acoustic sensor attached to the suprasternal notch to detect smoke related breathing.

Sensor modality: Smoke-related breaths can be detected by non-invasive acoustic sensors applied to the throat. Figure 7 illustrates the concept of external detection of breathing sounds.

Signal Processing and Pattern Recognition: A simple threshold-based classifier was presented by Echebarria et al. [57] for automatic identification of smoking breaths from acoustic signals. In Cui et al. [99], a hierarchical processing framework was proposed where the sub-movements of smoking from the recorded acoustic signal were detected in the lower level, and smoking puffs and sessions were detected on the higher level using a temporal sequence analysis technique. In both approaches [57,99], no de-noising method of the raw acoustic signal was explicitly mentioned.

Evaluation: The validation details of both approaches are presented in Table 11. The validation of the algorithm used by Echebarria et al. [57] involved six smoking events of 2 subjects in a lab environment, whereas the validation of [99] involved 143 smoking events of 16 subjects over a week of free-living conditions. The average recall of the algorithm used in [57] was 0.66 for the detection of smoking breaths. The algorithm applied in [99] achieved an accuracy F1-score of 0.93 and 0.92 in the detection of both the smoking puff and smoking frequency, respectively.

#### 4.1.6. Detection of Smoking Events Based on Egocentric Camera

**Figure 8:**
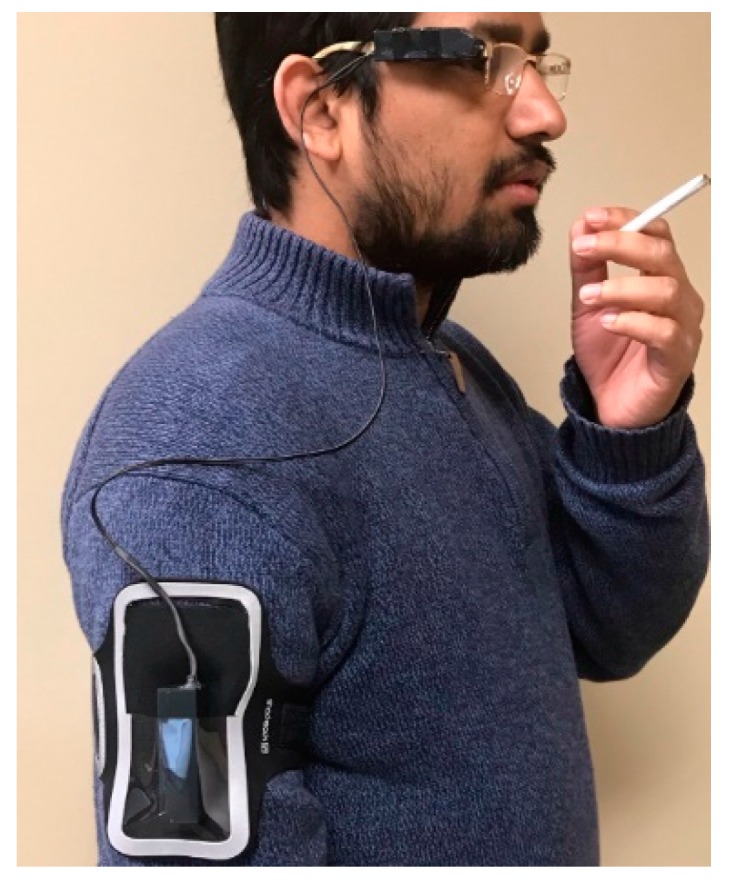
Egocentric camera attached to the eye-glass temple to captured details of smoking event.

Sensor modality: The smoking behavior can be monitored by a wearable egocentric camera, as the captured scenes contain details of the smoking event, smoking environment, body posture and activities during smoking. Figure 8 shows an egocentric camera-based sensor implementation.

In a paper under review [100], a laboratory test was reported for identifying the best position of camera placement on the body and maximizing the capture of images of smoking events. A low power, lightweight (11g) and tiny (6.5 × 1.9 × 1.5cm) sensor system were developed which composed of a wide-angle (120°) five-megapixel camera interfaced with an ultra-low-power microcontroller to capture and store a high resolution (2592 × 1944) image at every second up to 26 h on a single charge. Following the sensor placement test, the enclosure of the system was gaze-aligned with a provision on one side to facilitate the attachment to the temple of an eyeglass frame using 5mm double-sided acrylic tape. The DC supply of the wearable system was routed from outside the primary enclosure and placed into another enclosure and kept in an armband while wearing the sensor system.

Signal Processing and Pattern Recognition: Captured images by the egocentric camera were manually annotated in the paper under review [100] to obtain behavioral metrics of smoking, including daily smoking frequency, smoking environment and puffs per cigarette.

For an automatic extraction of behavioral metrics from captured images, two Faster R-CNN-based deep-learning models were developed and compared in the other paper under review [101] to (1) detect smoking events from images of lighting up a cigarette, and (2) smoking images that show a cigarette was being held in hand or mouth. Smoking information was next extracted from the detected smoking events, including the smoking time of day, frequency and inter-cigarette interval, etc.

Evaluation: The feasibility of the wearable egocentric camera was evaluated by a study performed on ten smokers to monitor full-day smoking under free-living conditions. Statistical tests (chi-square and t-test performed in the paper under review [100]) found significant differences between the information of smoking environment and puff count captured by the camera and self-reports. The computer model presented in the paper under review [101] for automatic detection of smoking events from cigarette lighting achieved higher F1-score (0.86) and recall (0.88) than the detection from smoking images (0.76 and 0.80, respectively). This study illustrated the applicability of camera-based wearable sensors for extracting an objective summary of daily smoking.

### 4.2. Multi-Sensor Fusion Approach

Generally, the fusion approach combines two or more wearable approaches in one platform to increase accuracy, synchronize utilization and simplify signal processing by eliminating the drawbacks of single-sensor approaches (presented in Table 12). Currently, three fusion sensor platforms have been introduced to enable the state-of-the-art research on smoking behavior. A summary of these platforms and the achievements of fusion approaches are presented below (with a comparison chart in Table 13):

#### 4.2.1. PACT

Sensor modality: The first evident fusion approach was the combination of breathing and hand gesture detection sensors in a platform called Personal Automatic Cigarette Tracker (PACT [90]). PACT contained RIP module respiratory bands, an RF proximity transmitter coil, a custom-made receiver antenna and a portable data logger. PACT was able to monitor smoking by following the characteristic hand-to-mouth gestures preceding the breathing patterns specific to smoke inhalations.

Signal Processing and Pattern Recognition: Several algorithms were proposed in a series of publications [102,103,104,105] based on the combination of RIP breathing and Proximity sensors of the PACT system. A comparison of these detection approaches is provided in Table 14.

Similar signal pre-processing steps were followed in these algorithms, including signal normalization and de-noising of tidal volume using a Gaussian average filter and a bandpass filter. Detection methods with all these approaches started by applying thresholds to detect HMGs, forming feature vectors from both sensor signals adjacent to the beginning of HMGs, and then applying machine learning classification methods, including SVM or ensemble algorithms, such as boosting (AdaBoost), bootstrap aggregating (bagging), and Random Forests.

Evaluation: The validity of the PACT system was tested by a study on 20 regular smokers performing 12 different activities in a controlled laboratory environment. The highest accuracy of the proposed algorithms for detecting hand to mouth gestures related to smoking was reported as 0.81 (F1-score) [103] for the subject-independent model and 0.94 (F1-score) for the subject-dependent model [103]. The minimum accuracies were reported as 0.41 [104] and 0.68 [103], respectively. In a comparative study [106], it was shown that the ensemble approaches outperformed the SVM classifier in both models. The “impact of subjects’ anthropometric characteristics on the quality of sensor signals” was evaluated by Patil et al. in [104], which found that subjects with medium BMI, high BMI, and standing posture were 1.91, 4.74 and 4.32 times more likely, respectively, to have their breathing signal quality affected.

#### 4.2.2. AutoSense

Sensor modality: AutoSense is an unobtrusive wearable wireless sensor suite [92] that measures several physiological and activity signals to monitor cardiovascular, respiratory and thermoregulatory systems. AutoSense combines six sensors into a wearable chest band—two ECG leads, a custom-made RIP band, a galvanic skin response (GSR) measurement system, a thermistor to measure skin temperature under the arm, an ambient temperature sensor, and a three-axis accelerometer to assess motion. AutoSense has been employed in a smoking study [107] with an additional wristband comprising a three-axis accelerometer, a 3-axis gyroscope, a 3-axis magnetometer, and two ambient-light sensors.

Signal Processing and Pattern Recognition: Data from the respiration sensor of AutoSense and wrist IMU were analyzed by Charles et al. in [88] with an algorithm tailored for recognizing smoking gestures with considerably reduced computational complexity. Preprocessing of the sensor data was done by outlier removal, i.e., discarding data when the sensors were not worn, and ignoring the segment of data packet loss in wireless transmission. From the preprocessed data, characteristic hand gestures were first identified; features were next computed from these hand gestures and corresponding respiration cycles; finally, an SVM was trained. A smoking episode was predicted in this algorithm if a cluster of smoking puffs were detected in the close temporal vicinity.

Evaluation: The validity of AutoSense sensors was tested on more than a hundred subjects both in a real-life behavioral science lab and field studies. The smoking detection algorithm proposed by Charles et al. [88] was validated on 40 h of data from six subjects in a laboratory environment. In a 10-fold cross-validation procedure, the proposed model achieved a recall rate of 0.96 and a false positive rate of 0.01 in detecting smoking events. This model was applied to three days of post-quit data from 32 smoking lapsers, and it correctly pinpointed the timing of the first lapse in 28 subjects. Only two false-positive episodes were detected during 20 days of abstinence. When tested on 28 subjects for the same 84 abstinent days, the occurrence of false episodes per day was limited to ~16%.

#### 4.2.3. PACT2.0

Sensor modality: An improved version of the PACT system called PACT2.0 was proposed by Imtiaz et al. [61], consisting of three devices: A miniature chest module to be placed at the center of the chest and attached to the RIP belt; a hand module to be worn on the wrist or forearm of the dominant hand; and an instrumented cigarette lighter. PACT2.0 contains several wearable sensors (Inertial, RIP, Electrical Proximity, physiological sensors such as Bioimpedance measurement and an ECG sensor) to monitor breathing, respiration rate, heart rate variability, hand-to-mouth gestures, posture and motion. In addition, PACT2.0 acquires and stores GPS location data, allowing the geospatial analysis of smoking events. Finally, the instrumented lighter keeps a log of all lighting events to increase the overall reliability of the system by an independent measurement of smoking behavior.

Signal Processing and Pattern Recognition: Senyurek et al. (Jan. 2019) [108] proposed an algorithm to identify the smoking session and smoking-related HMGs, integrating two PACT2.0 sensors: The instrumented lighter and the 6D wrist IMU. In the preprocessing steps, the raw IMU sensor signal was filtered by a 2nd order low pass Butterworth filter. Candidate HMGs were then detected from wavelet-filtered accelerometer 1D x-axis data, and an SVM classifier was trained. A two-level correction method was finally applied: A kernel-based smoothing and the identification of the start of the smoking session from the records of the instrumented lighter.

Using a similar combination of wrist IMU and instrumented lighter, Senyurek et al. (May 2019) [109] proposed to detect smoking events from the regularity of hand gestures estimated from a single axis of the accelerometer. In this approach, an unbiased autocorrelation method was applied to process the temporal sequence of hand gestures and to quantify the regularity score. A smoking episode (the start of smoking and duration) was predicted in this algorithm from lighting events and regularity scores.

In the paper under review [110], cycles of smoking inhalation were detected from RIP and IMU sensor signals employing deep learning models. CNN was first employed to automate feature learning from raw sensor streams. LSTM network layers were then used to capture the temporal dynamics of sensor signals and classify time segmented sequences.

Imtiaz et al. (2019) [111] proposed a smoking detection method using associated changes in metrics derived from the heart rate. To differentiate these changes from the impact of intense physical activities, the proposed system captured breathing (employing the bioimpedance sensor of PACT 2.0) and body motion (PACT 2.0 accelerometer placed on chest) along with heart rate (ECG sensor of PACT 2.0). A support vector machine-based classifier was developed on fifteen features of these sensor signals selected by a forward-feature selection method. A Gaussian kernel smoothing method was applied to the classifier outputs to identify the individual smoking session.

Evaluation: The smoking detection algorithms proposed in Senyurek et al. (Jan 2019) [108] were validated on 35 subjects in a controlled laboratory setting for three hours and free-living settings for ~24 h. In a leave-one-out-cross-validation on ~55 h of laboratory data, the combined approach [108] reached accuracies of 0.97 and 0.93, and F1-scores 0.98 and 0.86 for detecting smoking activity and individual HMGs, respectively. This model was also validated on ~816 h of free-living data with a reported accuracy of 0.82 in detecting smoked cigarettes in naturalistic settings. A comparable accuracy of 0.83 (F1-score: 0.91) was reported by Senyurek et al. (May 2019) [109] in identifying free-living cigarette smoking from the regularity of hand gesture and lighting events. Also, in Senyurek et al. (Jan. 2019) [108], authors identified that smokers under surveillance consumed cigarettes much faster (~5.6 min), with a higher number of hand to mouth gestures (15.04 gestures/cigarette) than under free-living conditions (~7.5 min, 8.9 gestures/cigarette).

The detection model of smoking inhalation presented in the paper under review [110] was evaluated on 467 free-living smoking events collected from 45 subjects over 42.7 h. This model achieved an F1-score of 0.78 in Leave-One-Subject-Out (LOSO) cross-validation.

In other LOSO validation, the physiological sensor-based approach [111] detected smoking events (187 out of total 232) with the recall and F1-score accuracy of 0.87 and 0.79, respectively, in the laboratory setting (known activities) and 0.77 and 0.61, respectively, under free-living conditions.

## 5. Discussion

This review was intended to provide a systematic evaluation of existing wearable sensors for the objective detection of behavioral and physiological manifestations associated with cigarette smoking. This review identified five specific phenomena related to cigarette smoking that were targeted in the development of wearable sensors. The review also explored 51 research publications describing methods to identify and evaluate smoking-related features assessed through individual sensor systems or their combinations.

The review found evidence that instrumented lighters can capture the initiation of a smoking sequence, and are capable of collecting data on smoking frequency in an unobtrusive way. Further, the lighter can be used in multi-sensor approaches for establishing the beginning of a smoking session. However, if the smoker uses a different lighter than this instrumented one, those particular smoking events will not be detected.

Studies covered in this review suggested that RF Proximity sensors can be effective tools for determining the frequency and duration of hand gestures preceding smoking. In a typical cigarette holding gesture, RF antennas were reported by Sazonov et al. [53] to produce the highest magnitude of signal strength relative to hand gestures associated with other activities (such as eating). However, these differences in signal amplitude may not be sufficient to differentiate among general hand-to-mouth gestures [63], but may be capable of providing supportive features to be used for the analysis of smoking patterns in multi-sensor approaches. Furthermore, this approach is typically used to detect gestures of the dominant hand; any smoking using a non-dominant hand (e.g., while driving) will also go undetected. The effectiveness of this method might be limited if a subject generates more frequent hand movements (not related to smoking) near the face. Also, the method will not be able to distinguish whether a person is smoking or resting/reading while supporting his chin with the smoking arm or hand.

IMUs were also found useful for detecting transitions of arm positions during smoking. Initial research with this approach involved the placement of multiple IMUs (3D or 6D) on different hand positions. However, recent research has focused on single IMUs. The 9D IMUs were also employed where concerns of battery longevity were not present. However, IMUs cannot provide information about the absolute position of the arm and its proximity to the mouth. The central challenge of the IMU-based approach is to recognize a smoking gesture ‘in the wild’ without any explicit information from the plethora of other gestures that a user performs each day. Furthermore, there are significant signal variations due to the changes in the users’ body orientation. When people swing their hands during smoking or in conversation, smoking hand gestures are difficult to identify. In some cases, wrist-worn sensors may not remain fixed in the initial placement position, and the sensor responses may vary under free-living conditions. Also, concurrent activities (e.g., walking, talking) while smoking modifies the characteristic pattern of the smoking gesture. Smartwatches also have these inherent limitations; however, they might facilitate real-time intervention (with or without pairing with smartphones).

RIP sensors are effective in capturing variations in the volume of inhaled smoke, the duration of inhalation and breath-holding time, and in bronchial reactivity. The breathing patterns measured through RIP sensors are highly susceptible to artifacts caused by hand and body motions. Stress, speaking, walking or other confounding events also had some effects on respiration measurements [91]. Processing of RIP sensor signals and robust classification algorithms are required to detect smoking patterns. RIP devices may also be cumbersome if worn for an extended period. Unlike initial implementations, recent RIP approaches contain miniature data logging modules with more comfortable elastic bands. Nevertheless, there are ongoing concerns and issues with clothing integration, cleaning and obtrusiveness of the devices.

Bio-impedance measurement systems are free of the limitations of integration with clothing, however, they require electrode attachment to the body. A combination of Bio-impedance based breathing sensor, ECG sensor and accelerometer placed on the chest was employed by Imtiaz et al. (2019) [111] for the detection of smoking events. However, the model suffered from many false positives, especially in free-living conditions. This study assumed that changes in heart rate parameters during the study period were either due to cigarette smoking or intense physical activities. Any physiological or ambient factors (with light physical activities) that could lead to a change in heart rate parameters might have caused false positives with this approach. Also, the impact of smoking on physiological signals or the influence of concurrent activities would likely vary greatly among people. Wattal et al. [112] presented textile electrodes and connectors, which can be evaluated to ease the data collection of the ECG and bioimpedance signals.

Smoking detection based upon acoustic signals is susceptible to ambient noise, hence robust signal processing methods for speech and artifact rejection are necessary for high accuracy. The visibility of this sensor system to others might limit the mass implementation of this approach.

Despite the limitations of wearable sensors and the failure of sensor systems to be 100% accurate in the detection of smoking events in all circumstances, extant systems have identified interesting smoking-oriented phenomena. For example, research using instrumented lighters substantiated the idea that smokers tend to overestimate their smoking consumption, and may be unaware of many instances of their daily smoking [12]. The instrumented lighter has also identified daily recurrent patterns of smoking incidents on an individual basis [12]. Work with the breathing sensor has verified that smoking displays a specific breathing pattern [58]. Further, the combination of breathing and proximity sensors identified individual traits in breathing patterns [102]. This combination of sensors also demonstrated that anthropometric characteristics (such as obesity, gestures) of the person affect the quality of smoking-specific breathing signals [104]. Finally, a combination of inertial sensors and instrumented lighters revealed that smokers under surveillance consume cigarettes much faster with a higher number of hand to mouth gestures than when in free-living conditions [108].

Additional successes with wearable sensors in smoking research are likely to be achieved if factors such as size and comfort of wearable systems, applicability in daily usage and inconspicuous monitoring are addressed. Due to their form factors, custom wrist-worn inertial sensors or smart-watches might be relatively easier to adopt for daily usage. RIP breathing or acoustic sensors tend to be obtrusive and somewhat cumbersome if worn for an extended period. Hence, the miniaturization and commercialization of these sensor systems will foster their acceptance by all types of smokers.

The camera-based system provides a direct way (from images of lit cigarettes) to detect smoking. Unlike other sensor systems, this sensor could identify the smoking environment, social interaction and locations that may promote smoking. This information related to activity and context during/immediately prior to smoking could play an important role in developing smoking intervention methods. The timestamp embedded in these images can provide additional information on the smoking time of day, duration and frequency. In the feasibility study involving 10 participants, all smoking events were correctly detected; however, it requires further validation, involving many participants of varying age and demographic profiles.

Most of the validation of the above-mentioned systems was limited to laboratory settings. These systems need systematic evaluation under extended free-living conditions. These evaluations need to gather more detailed information about intrusion and comfort. Also, these methodologies need to be tested for significantly longer periods of time (weeks/months) to fully examine their operation before they can be employed for general use.

The number of participant’s involvement varied between validation studies. Out of 51 reviewed articles, 40 studies involved more than 10 smoker subjects (two studies involving instrumented lighter, eight involving RF proximity sensor, 12 involving inertial sensors, 16 involving breathing sensor, one involving acoustic sensor, one involving egocentric camera). However, the remaining 11 studies had tests conducted in very small populations (less than 10, as low as two participants). Since the focus of this review was the presentation of all proposed sensors for smoking detection and characterization, no papers were excluded due to the small study size. However, the strength of the conclusions drawn from such studies is limited.

Individual smoking patterns can be influenced by a variety of factors such as location (e.g., smoking zones, automobiles), ambient conditions, and physical postures (walking, standing, sitting, and relaxing). However, no study on wearable sensors has systematically analyzed the impact of these contextual factors on smoking. Hence, the available mobility sensors in the PACT 2.0 platform (GPS or pedometer) can be evaluated to investigate their impact on improving the accuracy of current smoking detection methods.

Data recording is another important aspect of the available systems. Custom-made sensor systems have either onboard flash storage or the capability of wireless transmission to a nearby receiver or smartphone. In most of these approaches, data can only be accessed offline for computer analysis and cannot assist smokers to react immediately to their smoking situations. The smartwatch based IMU approach [65], [113], introduced methods to implement real-time detection algorithms at the smartphone to facilitate real-time interventions. The study reported by Skinner et al. [70] provided an approach to eliminate the necessity of smartphones and integrated everything into a single node (a wristwatch). McClernon and Choudhury [114] and Qin et al. [115] proposed methods to use only smartphone sensors (Wi-Fi, GPS, and Accelerometer) data to detect smoking events. These above-mentioned approaches may be capable of recruiting social support groups to inhibit smoking behavior. For more robust interventions, a blend of a Smartwatch platform with other fusion modules could be explored. These systems could even relate to devices on an Internet of Things (IoT) network to develop new intervention strategies.

## 6. Future Directions

This review demonstrates that the monitoring of cigarette smoking by wearable systems is still in an early stage of development and requires considerable research before it is suitable for general usage. No single sensor system provides a complete and accurate solution for the detection of smoking, the characterization of smoke exposure and other behavioral characteristics of smoking. This systematic review has addressed some successes of wearables in revealing interesting smoking-related phenomena. However, the review has identified a variety of challenges and obstacles to be addressed in future research.

First, no wearable sensor system reached an accuracy of 100% (even in controlled laboratory settings) in the detection of smoking-related features. Existing research targeted all major behavioral and physiological manifestations of cigarette smoking (e.g., lighting, hand gestures); however, body-worn or intraoral chemical sensors could be explored for the detection of smoking and the measurement of smoke exposure. Direct targeting of key chemicals, such as nicotine, may offer a universal approach for monitoring traditional and electronic cigarettes. To improve detection accuracy, further methodological improvements targeting signal processing and pattern recognition should be explored for the sensors currently in use.

Second, very few studies provide quantifiable evidence of user comfort, acceptability and adherence during the studies. These should be assessed in a standardized manner by developing a psychometrically validated questionnaire directed specifically to sensors for monitoring cigarette smoking. Future studies should pay special attention to the objective measurement of adherence, which is critical for the reliability of measurements. Additional sensors may need to be integrated into the wearable sensors, specifically with the purpose of identifying if the wearable is being used.

Third, most wearables have been tested in research settings, and only a few prototypes have been tested for accuracy or applicability under real-life conditions. The huge variability of unscripted human behavior and the impact of a myriad of contextual factors may present significant challenges to some of the sensor systems that test well in the laboratory. Future studies should focus on realistic evaluations under free-living conditions.

Fourth, many of the presented devices operate off-line. The development of real-time detection and notification capabilities may pave the way for the development of sensor-based smoking interventions.

## 7. Conclusions

This paper presents a systematic review of the state-of-the-art wearable technologies for an objective monitoring of smoking, a crucial process for timely interventions to curb smoking. Existing approaches were thoroughly examined in this review, and upcoming technologies were also identified. The review found that present-day research is now focusing upon improving accuracy, testing outside of restricted laboratory conditions, enhancing the comfort level of sensor systems, determining efficient classification methods, and improving signal processing procedures. If these existing challenges can be addressed, wearable sensors may substantially contribute to reducing the mortality rate due to cigarette smoking.

## Figures and Tables

**Table 1:** Inclusion and Exclusion Criteria for the review.

Inclusion Criteria	Exclusion Criteria
1. Articles published in peer-reviewed venues.	1. Articles that considered tobacco smoking, other than using cigarettes.
2. Articles published since 1990.	2. Papers not written in English.
3. Articles must address a certain combination of words, i.e., (cigarette smoking/ smoking detection) + (sensor/ wearable) + (validation/ participant/ subject / human study).	3. Detection system other than first smoke.
4. Portable systems with embedded wearable sensors.	4. Subjects under the age of 18 years.

**Table 2:** Summary of the publication related to classical methods for smoking detection.

Article Types	Total Articles
Articles describe the self-reporting of cigarette smoking	16
Articles describe CO-measurement and biomarker-based approaches	10
Articles describe wearable and surveillance-video camera-based approaches	5

**Table 3:** Articles on smoking detection employing wearable sensors targeting the behavioral-physiological manifestations of smoking.

Phenomena Used for Smoking Detection	Number of Published Papers						
	<2007	2007–2009	2010–2011	2012–2013	2014–2015	2016–2019	Total
Cigarette Packet	-	-	-	-	-	-	0
Lighting Event	-	-	-	1	1	2	4
Hand to Mouth Proximity	-	-	1	5	1	1	8
Smoking Hand Gestures	-	-	-	4	4	11	19
Smoking-specific respiration pattern	3	-	2	5	2	4	16
Breathing Sound	-	-	-	-	-	2	2
Egocentric Vision	-	-	-	-	-	2	2
Total Publications (By year)	3	-	3	15	8	22	51

**Table 4:** A summary of instrumented lighters employed to detect cigarette lighting events.

Type	Versions	Type	Lighting Mechanism	Features/Limitation	Microcontroller	Interface	Battery	Validation Study
Ubi-Lighter [60]	V1	Electric Coil	Slide Down Switch	Often hard to light up	Atmega32U2	Universal Serial Bus (USB)	200 mAh	3 subjects (11.36 ± 8.15 days), Free-living
	V2	Gas Lighter	Push Switch	One-time usage device	Atmega32U2	USB	30 mAh	8 subjects (11.36 ± 8.15 days), Free-living
	V3	Piezo-Ignition based	Push Switch	Contact-less data transmission via Bluetooth Low Energy (BLE)	Atmega32U2	USB, BLE	48 mAh	-
PACT [61]		Gas Lighter	Push Switch	Hall sensor based	MSP430G2452	USB	210 mAh	40 subjects (24 h each), Free-living

**Table 5:** Comparison of two versions of Radio Frequency (RF) Proximity sensors employed in smoking research.

**Ref**	**Transmitter Circuit**	**Receiver**	**Transmitter Antenna**	**Receiver Antenna**	**Data Storage**	**Validation Study**
[53]	Simple sine wave oscillator with a rectangular loop antenna	Large receiver module	40 × 15 × 5 mm, 860 uH ± 10%, 13 ohms (Sonmicro)	100 × 110 × 5 mm, 1080 uH, and 8.3 ohms (Sonmicro)	Logomatic V2.0, Sparkfun Electronics (commercial data-logger)	20 subjects in the lab
[61]	Tank circuit, opposite ends of the series antenna are connected to an MCU, two 180° phase shifted PWM outputs (50% duty cycle)	Compa-ratively small receiver module	7.2 mH ± 2%, 91-ohm transponder coil (Coilcraft)	7.2 mH ± 2%, 91-ohm transponder coil (Coilcraft)	Embedded data logger with STM32 MCU	40 subjects both in the lab and free-living

**Table 6:** Comparison of Inertial sensors employed in smoking research. IMU = Inertial Measurement Unit.

Ref	IMU Type	Sensor Chip	Employed IMU Range	MCU	Sampling Frequency	Data Access	Validation Study
[54]	3D	ADXL345 on the ‘Hedgehog’ platform	± 2g	PIC18F	20 Hz	Embedded SD card	4 subjects
[69]	6D	MMA7260Q on the ShimmerTM Platform	± 6g and ± 500 degree/s	ShimmerTM Platform	50 Hz	Wirelessly Transmitted	6 subjects
[70]	9D	MPU-9150	-	-	50 Hz	Wirelessly Transmitted	19 subjects
[61]	6D	LSM6DS3	± 8g and 2000 dps	STM32L151RD	100 Hz	Embedded SD card	40 subjects

**Table 7:** Signal Processing and Pattern recognition techniques applied to the Inertial Measurement Unit (IMU).

Ref	IMU Type	Pre-processing	Candidate Selection	Window Size	No of Extracted Feature	No of Selected Feature	Classifier	Detection	Validation
[54]	3D	equalized ripple (equi- ripple) FIR low-pass filter (fc = 1 Hz)	Y-axis accelerometer	5.4 sec	4	4	Gaussian Mixture	Smoking	K-fold validation
[64]	3D	-	RF threshold	25 sec 50% overlap	5	5	Random Forest (RF), Thresholding	Hand-to-mouth gesture (HMG), Smoking	5-fold
[69]	6D	low-pass filter (fc = 5 Hz)	Moving window	10 sec	10	10	Support- vector machine (SVM), Edge detector	HMG, Smoking	-
[70]	9D	-	Distance calculation Moving window	-	34	34	Conditional Random Forest	HMG, Smoking	10-fold & leave one out cross validation (LOOS)
[66]	6D in smartwatch	-	Moving window	30 sec	6	4 (Empirically chosen)	Hierarchical 2 layer	Smoking	LOOS
[65]	3D in smartwatch	-	Euler transformation	-	3	3	Artificial Neural Network	Smoking	K-fold validation
[68]	6D in smartwatch	-	Hand movement	-	3	3	3 stage analytical pipeline using Decision Tree	Smoking	LOOS
[72]	3D in smartwatch	-	sliding window x-axis accelerometer	10 s	1	1	Dynamic Time wrapping algorithm (CWRT)	Smoking	LOOS

**Table 8:** Summary of detection algorithms employed on inertial sensors.

Ref	No of IMU	IMU Placement	Dataset	Subject	Activities	Study Type	Detection	Performance
[54]	1 (3D)	wrist	Data of 23 days	4	Smoking-standing	Free- living	Smoking	Precision 0.51, Recall 0.70
[64]	4 (3D)	Dominant wrist and upper arm, non-domin-ant wrist, ankle	11.8 Hour (34 smoking, 481 puff)	6	Smoking-eating, walk, Talk, Drink, Stand	Lab	HMG, Smoking	F1-score 0.70 for HMG, 0.79 for smoking
[69]	4 (6D)	Wrist, upper arm near the shoulder, upper arm near elbow, elbow	21 Hour	6	Smoking-sitting, walk, Smoking-resting, cellphone use	Lab	HMG, Smoking	False Positive Rate 0.07–0.2
[70]	1 (9D)	Wrist, elbow	28 Hour, 369 puffs (48 h for wild)	15-lab, 4-free-living	Smoking-stand, Smoking-talking, Smoking-walking, eat, drink	Lab, Free-living	HMG, Smoking	F1-score 0.85, Precision 0.95, Recall 0.81
[66]	1 (6D) in Smart watch	wrist	45 Hour, 17 h smoking of 230 cigarettes	11	Smoking-stand, Smoking-sitting, Eat, Drink, Group conversation, Sitting,	Lab	Smoking	F1-score 0.83–0.94 (person-independent) F1-score 0.90–0.97 (person-dependent)
[65]	1 (3D) in smartwatch	wrist	35 smoking, 155 non-smoking sessions,	2	Not mentioned	Lab	Smoking	Accuracy 0.85–0.95
[67]	1 (6D) in band	wrist	1584 epochs of hand gestures	1	Sitting, Walking, Eating	Lab	Smoking	Accuracy 0.94 Recall 0.91
[68]	1 (6D) in smartwatch	wrist	-	38	Smoking-sitting, Drink, Eat	Lab, Free-living	Smoking	Precision 0.86, Recall 0.71
[72]	1 (3D) in smartwatch	wrist	-	26	Smoking-stand, Eat, Drink	Lab	Smoking	F1-score 0.96

**Table 9:** A summary of wearable Respiratory Inductance Plethysmography (RIP) sensors employed in smoking research.

Ref	Belt Placement	RIP Belt/ Module	Signal Output	Data storage	Validation Study
[85]	Thoracic and abdominal	DuraBelt Pro-Tech Inc. connected to zRIP, Philips Respironics, Murrysville, PA	Analog Data	Commercial data logger: Logomatic V2.0, Sparkfun Electronics	20 subjects in the lab
[86]	Thoracic	AutoSense RIP belts	Analog Data	Wireless transmission to smartphone	35 in lab and free-living
[61]	Thoracic	SleepSense Inductive Plethysmography, S.L.P Inc.	Pulse Data	Embedded data logger with STM32 MCU	40 both in the lab and free-living

**Table 10:** A summary of detection algorithms employed on RIP sensors.

Ref	No of RIPBand	Pre-processing	De-noising	Artifact Removal	Feature Extracted	Classifier Employed	Signal Classification	Validation	Study Type	Performance Matrices
[89]	2 (Thoracic TC, Abdominal AB)	1. Tidal Volume and Airflow measurement from TC, AB signals 2. Signal Normalization to the range of -1 and 1	-	An ideal band pass filter, fc = 0.0001–10 Hz	-	Simple Peak-Valley Detection	4 activities (resting, reading aloud, food intake and smoking)	Train- 5 fold cross-val; Test-LOOS	Lab, 20 subject	Accuracy: Resting-0.96, Reading-0.89, Food intake-0.91, Smoking-0.89
[90]			Average Gaussian filter of 25 points		Z-norm 16 features Using Window 0.5s, 50% overlap	Left-to-right hidden Markov models	5 activities (sedentary, walking, eating, talking, and cigarette smoking)	LOOS	Lab, 20 subject	Precision 0.60, Recall 0.67 F1-score 0.62
[86]	1 (Thoracic TC)		-	-	17 features from each 30s window	Supervised and semi-supervised support vector	Puff or non-puff	LOOS	Lab, 10 subject	Accuracy 0.91

**Table 11:** A summary of acoustic sensor and validation study involved in smoking detection.

Ref	Sensor Details	Subject Involved	Study Details	Total Smoking Events
[57]	WADD 3,74 × 2.4 × 2.1 cm, 17 g	2	In lab (1 session)	6
[91]	Smart neckband: dual-core 1.5 GHz CPU, 1 GB RAM, Android 4.2 OS	16	Free-living (1 week)	143

**Table 12:** The comparison of key sensing modalities employed in smoking research.

Features of Wearable Systems	Respiratory Inductance Plethysmography	Electrical Proximity Sensing	Inertial Approach	Egocentric Camera
Body Positions	Abdominal or Thoracic area	Transmitter on wrist and Receiver on the chest surface	Mostly on wrist or lower elbow	Eye, chest or Wrist. Eye-level camera was explored.
Comfort	Moderate, worn as a belt	Moderate	High, flexible to implement in body locations	High, however a privacy concern exists
Applications	Characteristic breathing pattern detection	Characteristic hand to mouth proximity	Characteristic hand gesture of smoking	Smoking puff, environment, context detection
Highest Performance	Accuracy of 0.81 in detecting puff events [86]	Recall of 0.90 in detecting hand to mouth gestures preceding smoking [63]	Precision 0.95 and F1-score 0.85 in detecting smoking events [70]	Recall of 1 in detecting smoking events (manual image review)
Advantages	Indirect monitoring	Good tolerance to electromagnetic interference	Able to be embedded in a highly wearable wristband or smartwatch	Direct monitoring
Challenges	Accuracy needs improvement	Combination of other sensors is necessary to improve applicability	Detected gestures often confused with eating; limited by concurrent activity and confounding gestures	Privacy concern for both wearer and people in surroundings
Applicable to free-living settings	Thoroughly tested	Moderately tested	Thoroughly tested	Feasibility tested
Obtrusiveness	Unobtrusive	Unobtrusive	Unobtrusive	Unobtrusive
Contact with Skin	Not mandatory, can be worn over clothing	Not required	Not required if wristband employed	Not required.

**Table 13:** The comparison of multi-sensor approaches on a fusion platform.

Fusion Platform	AutoSense	PACT	PACT v2
Sensing element employed in smoking research	RIP sensing, 6-axis IMU (other sensors not utilized yet in smoking research)	RIP, Proximity	RIP, Bioimpedance sensor, ECG, 6-axis IMU and Instrument lighter (other sensors not utilized yet in smoking research)
Sampling Frequency	21.3 Hz for RIP, 16 Hz for Inertial Sensor	100 Hz	100 Hz for IMU, RIP, Proximity; 1 KHz for Physiological sensor
Device Storage	N/A	Portable Datalogger (Logomatic V2, Sparkfun Electronics, Boulder, CO)	On Board 4-GB Micro SD card
Sensor data Transmission Method	To smartphone via ANT Radio.	N/A	N/A
Data analysis/processing method	Published	Published	Published
Clinical or Validation Survey	Performed over more than 100 subjects in different studies	Performed over 20 regular smokers.	Performed over 40 regular smokers.
Tested in Free-living	Tested over 61 regular smokers in different studies	Not tested	Tested over 40 regular smokers.
Gold Standard Comparison	Manual annotation by an observer.	Push Button based manual annotation	Manual Video Annotation and cellphone registration
System longevity (Battery Life)	More than a day	More than a day	More than a day

**Table 14:** Summary of detection algorithms employed on the combination of respiration and proximity sensors.

Ref	De-noising and Artifact Removal	Pre-processing	Approach	Key Points	Performance Matrices		Validation
					Subject-Independent	Subject-Dependent	
[92]	**1. Gaussian Average Filter of 25-point sliding window** **2. Ideal Band Pass Filter: fc = 0.0001–10 Hz**	**Normalization on both Proximity Signal and tidal Volume**	SVM	**-**	Precision 0.87, Recall 0.80	Precision 0.90, Recall 0.90	LOOS
[93]			SVM	1503 Feature Vectors	F1-score: 0.81	F1-score: 0.90	LOOS
				27 Empirical Feature Vectors	F1-score: 0.65	F1-score: 0.68	
				16 Forward Feature Selected Feature Vectors	F1-score: 0.67	F1-score: 0.94	
[94]			SVM	Employing Thoracic Signal (TC)	F1-score: 0.41	F1-score: 0.85	LOOS
				Employing Abdominal Signal (AB)	F1-score: 0.46	F1-score: 0.88	
				Employing Proximity Signal (PS)	F1-score: 0.59	F1-score: 0.90	
[93]			Ensemble	Adaboost	F1-score: 0.71	F1-score: 0.77	LOOS
				Bagging	F1-score: 0.70	F1-score: 0.82	
				Random Forest	F1-score: 0.69	F1-score: 0.84	
